# Online searches for SGLT-2 inhibitors and GLP-1 receptor agonists correlate with prescription rates in the United States: An infodemiological study

**DOI:** 10.3389/fcvm.2022.936651

**Published:** 2022-07-29

**Authors:** Omar Dzaye, Philipp Berning, Alexander C. Razavi, Rishav Adhikari, Kunal Jha, Khurram Nasir, John W. Ayers, Martin Bødtker Mortensen, Michael J. Blaha

**Affiliations:** ^1^Johns Hopkins Ciccarone Center for the Prevention of Cardiovascular Disease, Johns Hopkins University School of Medicine, Baltimore, MD, United States; ^2^Department of Medicine, University Hospital Muenster, Münster, Germany; ^3^Emory Center for Heart Disease Prevention, Emory University School of Medicine, Atlanta, GA, United States; ^4^Division of Cardiovascular Prevention and Wellness, Department of Cardiology, Houston Methodist DeBakey Heart and Vascular Center, Houston, TX, United States; ^5^Division of Infectious Diseases and Global Public Health, University of California, San Diego, CA, United States; ^6^Department of Cardiology, Aarhus University Hospital, Aarhus, Denmark

**Keywords:** cardiometabolic medicine, SGLT 2 inhibitors, GLP-1 receptor agonist, Google trends, national prescription audit

## Abstract

Several clinical trials have demonstrated that many SGLT-2 inhibitors (SGLT2i) and GLP-1 receptor agonists (GLP-1 RA) can reduce the risk of cardiovascular events in patients with Type 2 diabetes and atherosclerotic cardiovascular disease. Recent reports indicate an underutilization of new cardiometabolic drugs, including SGLT2i and GLP-1 RA. We aimed to evaluate the use of online search volumes to reflect United States prescription rates. A repeated cross-sectional analysis of Google search volumes and corresponding data from the IQVIA National Prescription Audit (NPA) of pharmacy dispensing of newly prescribed drugs was performed. Monthly data for online searches and prescription between January 1, 2016 and December 31, 2021 were collected for selected SGLT2i and GLP-1 RA. Prescription data for drugs classes (SGLT2i and GLP-1 RA) and individual drugs were calculated as the total of queried data for branded drug names. Trends were analyzed for visual and quantitative correlation as well as predictive patterns. Overall, online searches increased by 157.6% (95% CI: 142.2–173.1%) and 295.2% (95% CI: 257.7–332.6%) for SGLT2i and GLP-1RA between 2016 and 2021. Prescription rates raised by 114.6% (95% CI: 110.8–118.4%) and 221.0% (95% CI: 212.1–229.9%) for SGLT2i and GLP-1RA for this period. Correlation coefficients (range 0.86–0.99) were strongest for drugs with growing number of prescriptions, for example dapagliflozin, empagliflozin, ertugliflozin, dulaglutide, and semaglutide. Online searches might represent an additional tool to monitor the utilization trends of cardiometabolic drugs. Associations were strongest for drugs with reported cardioprotective effect. Thus, trends in online searches complement conventionally acquired data to reflect and forecast prescription trends of cardiometabolic drugs.

## Introduction

Cardiovascular disease (CVD) represents the leading cause for mortality and morbidity among patients with type 2 diabetes (T2D) (1). This is of particular relevance to public health in the United States since the rates of adults with pre-diabetes are constantly rising and accounting for more than one third of the adult US population (2). In the last seven years, clinical trials have demonstrated that many SGLT-2 inhibitors (SGLT2i) and GLP-1 receptor agonists (GLP-1 RA), which were both initially approved as glucose-lowering medications, can reduce the risk of cardiovascular events in patients with T2D and established atherosclerotic CVD (3, 4). Based on these results, FDA labels and guideline recommendations for several of these drugs were expanded to reflect on the CVD-risk reduction in T2D patients (5, 6). However, recent reports indicate a delayed adoption of SGLT2i and GLP-1 RA medications in eligible patients throughout the United States (7, 8).

Online search behavior has become an increasingly valuable measure for public health trends. Given the ubiquitous availability of online search tools and the near-real time accessibility of the respective search data allows to monitor and potentially forecast patient behavior in various health-related issues (9, 10). Google search data as the most used search engine worldwide are publicly accessible through the Google Trends tool. Several studies indicate that Google searches can temporarily align with conventional epidemiologic parameters, correlate with geographical trends and thus potentially forecast trends in for example acute coronary syndrome admissions, cardiovascular disease, and mental disorders (11–15).

Given the delays in the adoption of SGLT2i and GLP-1 RA as drugs that can improve cardiometabolic outcomes, we aimed to provide a comparative overview of online search activity and prescription rates of these cardiometabolic drugs. We hypothesized that trends in online search behavior could mirror prescription activity and eventually can provide a near-real time tool to forecast future trends in the adoption of these drugs with relevance to the field.

## Materials and methods

### Data collection

Monthly search data for the United States were retrieved for the period between January 1, 2016 and December 31, 2021 using Google Trends for Health Application Programming Interface. Online search data were extracted from Google Trends as query fractions per 10 million searches for drugs representative of the SGLT2i and GLP-1 RA drug classes. Additionally, online search data for Biguanide (metformin) and Sulfonylureas (glimepiride, glipizide, glyburide) were extracted. All queried search terms are summarized in Supplementary Table 1. In a next step, queries were aggregated for overall trends in drug classes and for individual drugs. Individual drug analyses considered brand names, which accounted for the majority of searches, and chemical names as well as the combination or brand name plus chemical names.

Drug prescription data were extracted from the IQVIA National Prescription Audit (NPA). The NPA provides a measure of overall national prescription dispensing information from outpatient pharmacies. These data include prescription data from more than 70% of all prescription activity in the United States and are then projected to estimate 100% of prescriptions. Prescriptions from all payers including Medicare and Medicaid are captured. Further detailed information on the data collection process can be found elsewhere (16). We queried monthly prescription data between January 1, 2016 and December 31, 2021 for selected SGLT2i, GLP-1 RA, Biguanide (metformin) and Sulfonylureas brands as listed in Supplementary Table 1. Prescription data for drugs classes and individual drugs were calculated as the total of queried data for branded drug names. We used monthly data for newly dispensed prescriptions as a fraction of total monthly prescriptions (per 10 million prescriptions) to optimize comparative analyses with online search data.

### Statistical analysis

To compare search trends, Microsoft Excel, version 16.52, was used to compute time-averaged monthly searches and prescriptions rates. Calculations and data visualization, including Spearman's correlation for non-parametric variables between monthly prescriptions and online searches, were performed in Microsoft Excel and GraphPad Prism 9.0 software. An autoregressive integrated moving average (ARIMA) model was applied to forecast current trends in prescription rates with January 1, 2021 as a cut-off. ARIMA calculation was performed using the gtrendR package in R software, version 4.1.1 (R Foundation for Statistical Computing).

## Results

**Figure 1:**
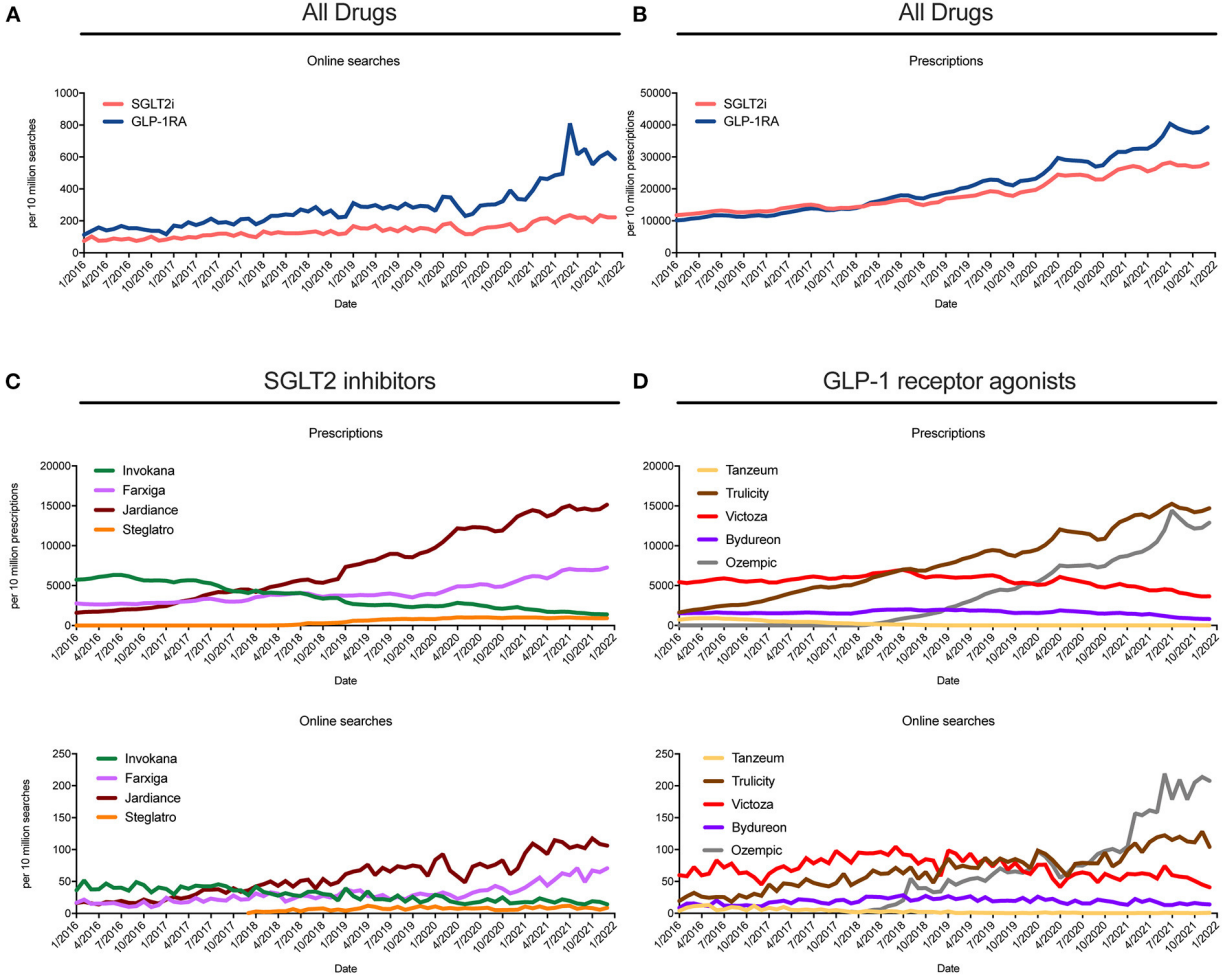
Trends in prescriptions and online searches for SGLT2i and GLP-1 RA brands between 2016 and 2021 for the United States. **(A)** Online searches and **(B)** prescriptions from 2016 to 2021 for SGLT2i (red line) and GLP-1 RA (blue line) as monthly query fraction/prescriptions per 10 million searches/prescriptions for summarized brands names are shown. **(C)** Trends in prescriptions (upper panel) and online searches (lower panel) for 4 selected SGLT2i brand names. **(D)** Trends in prescriptions (upper panel) and online searches (lower panel) for 5 selected GLP-1 RA brand names. All data are representative for the United Sates.

Prescription and corresponding online search data were gathered for five SGLT2i and four GLP-1 RA as prescriptions or online searches per 10 million between 2016 and 2021. As shown in Figures 1A,B, online searches and prescription rates showed a similar increasing trend for both drug classes. Overall, for SGLT2i, online searches increased by 157.6% (95% CI: 142.2–173.1%) and prescription rates by 114.6% (95% CI: 110.8–118.4%) between 2016 and 2021. For GLP-1 RA, increases of 295.2% (95% CI: 257.7–332.6%) for online searches and 221.0% (95% CI: 212.1–229.9%) for prescriptions were noted between 2016 and 2021. Next, trends in prescription rates and online searches for the respective brands with the highest prescription rates grouped by SGLT2i and GLP-1 RA are depicted in Figures 1C,D. For the individual SGLT2i brands Jardiance^®^ (empagliflozin) and Farxiga^®^ (dapagliflozin), trends in prescriptions and online searches showed a nearly identical increase. For Steglatro^®^ (ertugliflozin) a less strong increase could be noted, while for Invokana^®^ (canagliflozin) a decreasing trend was observed (Figure 1C). The selected GLP-1 RA panel revealed clear increases in both prescriptions and online searches for Trulicity^®^ (dulaglutide) and Ozempic^®^ (semaglutide), while for Victoza^®^ (liraglutide), Bydureon^®^ (exenatide) and Tanzeum^®^ (albiglutide) prescription rates and online searches remained constant with lowest rates for Tanzeum™, a drug which was removed from the market in 2018 for commercial reasons (albiglutide) (Figure 1D).

**Figure 2:**
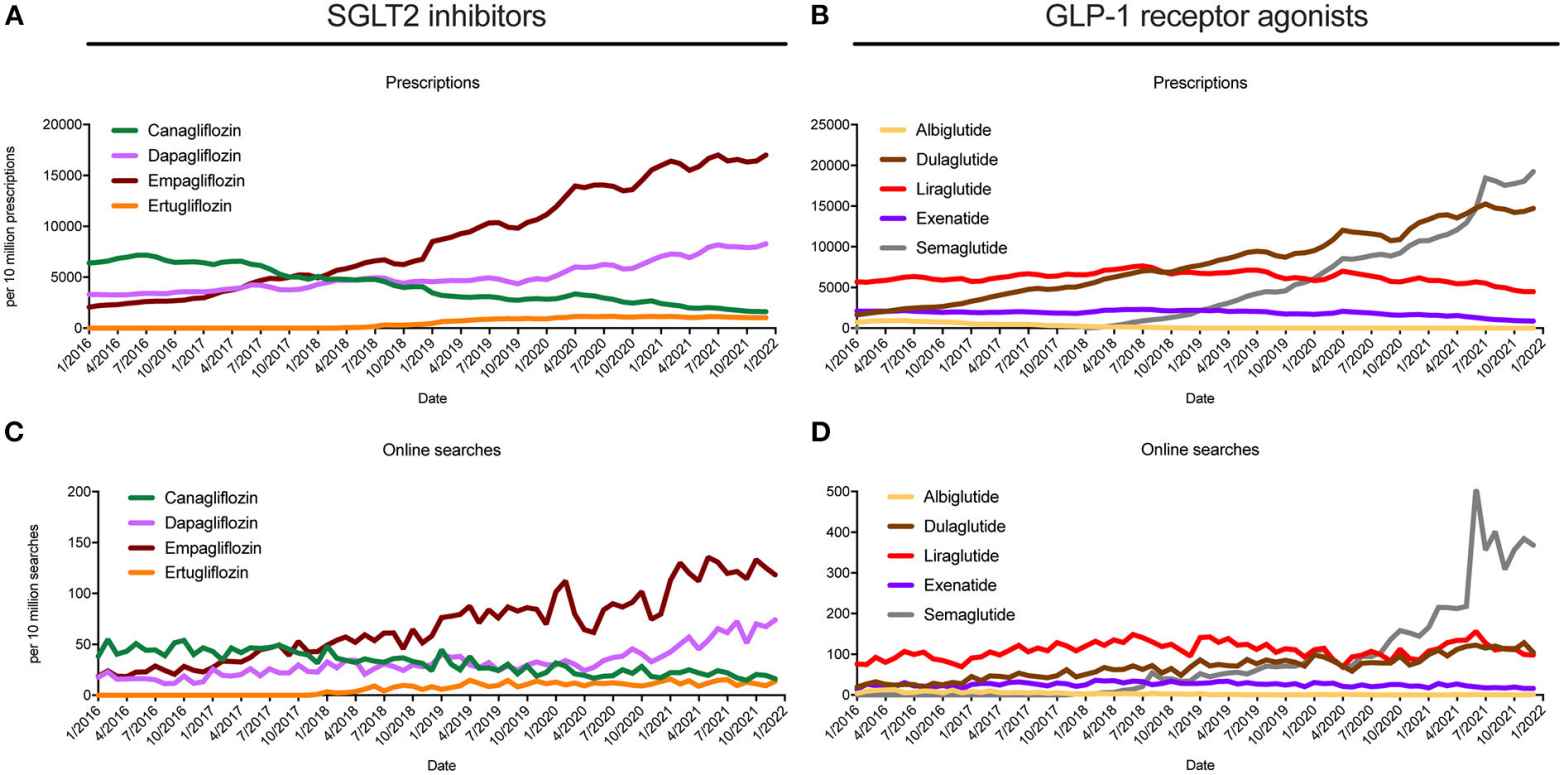
Trends in prescriptions and online searches for SGLT2i and GLP-1 RA drug names between 2016 and 2021 for the United States. Prescription rates for selected **(A)** SGLT2i and **(B)** GLP-1 RA drug names as monthly prescriptions per 10 million prescriptions are shown. Trends in online searches for **(C)** SGLT2i and **(D)** GLP-1 RA are depicted as monthly query fraction per 10 million searches. All data are representative for the United Sates between 2016 and 2021.

These trends in online searches and prescriptions were similarly observed for the individual drugs, which are shown in Figure 2. For dapagliflozin, empagliflozin, and dulaglutide online searches increased by 300.1% (95% CI: 225.6–374.6%), 444.6% (95% CI: 407.6–481.6%) and 346.5% (95% CI: 297.2–395.8%) between 2016 and 2021, respectively. Prescriptions rates showed differences of 126.5% (95% CI: 121.4–131.7%), 559.0% (95% CI: 525.1–593.0%), and 525.2% (95% CI: 467.7–582.6%) during this period. For ertugliflozin and semaglutide, prescriptions and online searches were only detectable beyond January 2018, with differences in online searches of 166.4% (95% CI: 75.8–256.9%) for ertugliflozin and 2,879.9% (95% CI: 1,306.3–4,453.6%) for semaglutide between 2018 and 2021. While, in parallel, prescription rates for ertugliflozin increased by 4,086.4% (95% CI: −738.8 to 8,911.6%) and for semaglutide by 3,618.6% (95% CI: 997.5–6,239.8%). In contrast, for Canagliflozin, Albiglutide, and Exenatide, both prescriptions and online searches showed an overall decrease, while for Liraglutide no significant differences between 2016 and July 2021 were noted (Table 1).

**Table 1:** Differences between 2016 and 2021 in prescriptions and online searches of SGLT2i and GLP-1 RA.

**Drug/Brand name**	**Prescriptions** **2016 vs. 2021** **changes in % (95% CI)**	**Online searches** **2016 vs. 2021** **changes in % (95% CI)**
**SGLT-2 inhibitors**		
Dapagliflozin	126.5 (121.4 to 131.7)	300.1 (225.6 to 374.6)
FARXIGA	143.8 (135.1 to 152.5)	313.9 (230.8 to 397.0)
QTERN	*	*
XIGDUO XR	55.8 (44.2 to 67.3)	108.5 (2.7 to 214.3)
Canagliflozin	−71.1 (−73.5 to −68.7)	−54.3 (−60.0 to −48.6)
INVOKAMET	−66.7 (−71.3 to −62.2)	−34.1 (−70.8 to 2.7)
INVOKANA	−71.7 (−73.8 to −69.5)	−54.6 (60.5 to −48.8)
Empagliflozin	559.0 (525.1 to 593.0)	444.6 (407.6 to 481.6)
JARDIANCE	657.7 (614.2 to 701.1)	516.9 (489.8 to 544.0)
SYNJARDY	*	*
GLYXAMBI	−11.3 (−16.7 to −5.9)	−1.8 (−43.0 to 39.5)
TRIJARDY	*	*
Ertugliflozin	*	*
SEGLUROMET	*	*
STEGLATRO	*	*
STEGLUJAN	*	*
GLP-1 receptor agonists		
Albiglutide	−100.0	−94.3 (−97.9 to −90.7)
TANZEUM	−100.0	−94.3 (−97.9 to −90.7)
Dulaglutide	525.2 (467.7 to 582.6)	346.5 (297.2 to 395.8)
TRULICITY	525.2 (467.7 to 582.6)	346.5 (297.2 to 395.8)
Liraglutide	−11.7 (-17.5 to −5.9)	38.6 (29.8 to 47.3)
VICTOZA	−23.2 (−28.4 to −18.0)	−10.8 (−16.5 to −5.1)
SAXENDA	157.8 (129.6 to 185.9)	183.4 (154.7 to 212.1)
Exenatide	−40.2 (-47.2 to −33.2)	−2.9 (−13.8 to 8.1)
BYDUREON	−27.1 (−36.8 to −17.4)	28.8 (13.2 to 44.4)
BYETTA	−81.4 (−82.1 to −80.7)	−51.1 (−61.7 to −40.4)
Semaglutide	*	*
OZEMPIC	*	*
RYBELSUS	*	*
WEGOVY	*	*

CI, confidence interval; * no prescription/online search data available for 2016 as baseline.

**Figure 3:**
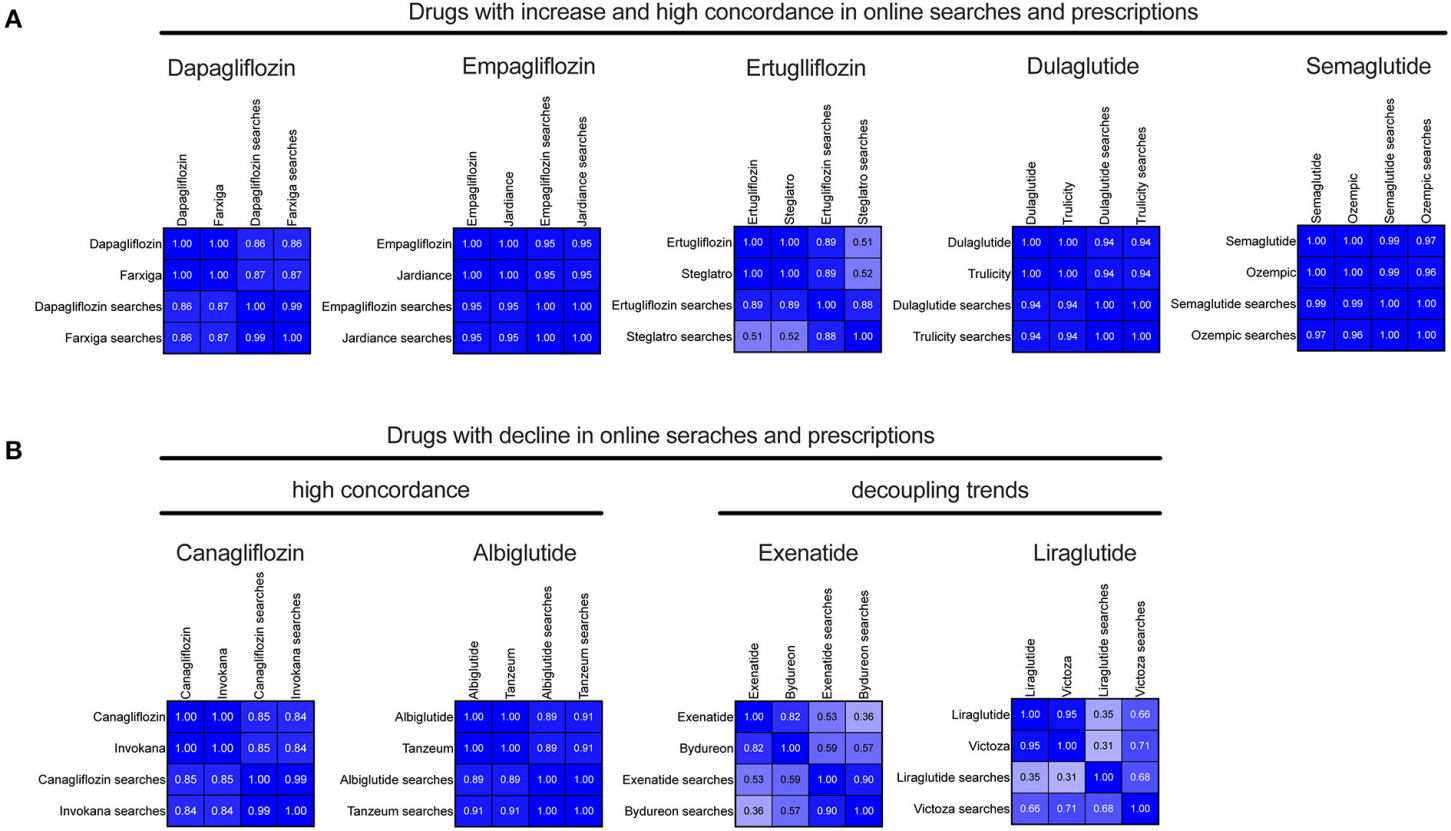
Correlation analyses between prescriptions and online searches for SGLT2i and GLP-1 RA. Correlation matrices for **(A)** drugs and corresponding brand names with increasing trends in both prescriptions and online searches (indicated by “searches”). **(B)** Drugs and corresponding brand names with overall decreasing trends in both prescriptions and online searches (indicated by “searches”). Spearman's correlation coefficients were calculated for the period between 2016 and 2021.

For the drugs with an overall increase, namely dapagliflozin, empagliflozin, ertugliflozin, dulaglutide and semaglutide, online searches and prescriptions showed strong correlation coefficients of 0.86 (95% CI: 0.79–0.91), 0.95 (95% CI: 0.92–0.97), 0.89 (95% CI: 0.83–0.93), 0.94 (95% CI: 0.90–0.96), and 0.99 (95% CI: 0.98–0.99), respectively (Figure 3A). For drugs with a decreasing usage trend, a high concordance of online searches and prescriptions was observed for canagliflozin and albiglutide, while for exenatide and liraglutide a decoupling of usage trends was detected (Figure 3B). Correlations for brand names and online searches revealed comparable results (Figures 3A,B). In an additional autoregressive analysis encompassing drugs with growing trends, prescriptions rates after January 1, 2021 can be generally forecasted using the recent historical trends in online searches (Supplementary Figure 1). Additionally, prescription and online search trends for other exemplary antidiabetic drug classes, notably biguanide (metformin) and sulfonylureas, are shown in Supplementary Figure 2. While overall online searches between 2016 and 2021 remained flat or constant for sulfonylureas and modestly increased for metformin, prescription slightly decreased (sulfonylureas) or remained constant or slightly increased for metformin, consistent with modest correlation for these older classes of antidiabetic drugs.

## Discussion

The present study shows a strong correlation between online searches and actual prescription rates for SGLT-2 inhibitors and GLP-1 receptor agonists from 2016 to 2021 in the United States. In particular, correlations between online search volume and prescriptions were strongest for newer drugs with increasing prescription rates, as well as those with sudden shifts toward decreasing rates due to warnings or withdrawal from the market, while a sudden and rapid decoupling was noted for older GLP-1 receptor agonists that were subsequently shown to have decreasing prescription rates. These results have implications for rapid evaluation of changes in attitudes and behaviors toward cardiometabolic drugs in the United States.

To our knowledge, our study is the first to evaluate the potential use of online search monitoring to forecast prescription trends for drugs that are relevant to cardiometabolic medicine, and particularly the combined management of CVD and T2D. Although there is growing evidence for the use of both SGLT2i and GLP-1 RA in T2D patients with established CVD and/or CVD risks, prescription rates indicate that both drug groups are yet not made accessible to most patients with T2D and CVD (7, 17). Our data show that the monitoring of online search interests might provide a near-real-time and accessible tool to monitor perturbations and forecast the actual adoption by healthcare professionals and their patients.

For the drugs with high correlations between online searches and prescriptions, dapagliflozin, empagliflozin, ertrugliflozin (SGLT2i) and dulaglutide, semaglutide (GLP-1 RA), have some of the strongest and most recent data for cardioprotective effects across several trials (3, 18). Ertugliflozin and semaglutide received the first FDA approval for T2D treatment as early as late 2017, which might indicate for an increased awareness for these drugs (19, 20). Our data showed tight correlations between online searches and decreasing prescription rates for canagliflozin that immediately followed the warnings for lower extremity amputation (21). Albiglutide was withdrawn from the market in 2017 which corresponds well with the prescription trends. Taken together, these findings indicate that trends in online searches are also sensitive to sudden decreases in prescriptions. Whereas for drugs with lower correlation levels overall limited changes and gradually decreasing trends in utilization and searches between 2016 and 2021 were noted. Examples of this include exenatide, for which cardioprotective effects are less clear, and the cardioprotective drug liraglutide, for which prescriptions may have shifted towards semaglutide because of the daily vs. weekly dosing and marketing efforts from the manufacturer (3).

Our study has limitations. First, prescription data for newly generated prescription numbers were considered, which include both newly prescribed drugs as well as refills or continuation of existing medications with a new prescription number. Therefore, the present data cannot represent patients already taking SGLT2i or GLP-1 RA but with a continuing prescription number. However, this method ensures that the recorded prescriptions include all patients newly receiving the drugs and makes the analyses more sensitive to changes in prescribing trends. Second, Google Trends online search data solely represents searches on Google and affiliated services. As a result, younger and more tech-savvy individuals seeking health-related information might be overrepresented by Google Trends. Finally, the identity of those conducting online searches (patients, clinicians, media, etc.) are unknown. However, this reflects the core hypothesis being tested—that the totality of online interest displayed toward a drug reflect present and potentially future prescribing behavior.

Overall, we observed a strong correlation between online searches and prescription rates for SGLT-2 inhibitors and GLP-1 receptor agonists in the United States, and we demonstrated the feasibility of forecasting future prescription rates using online search rates. Associations were strongest for drugs with reported cardioprotective effects. Therefore, online searches might represent an additional tool to monitor the utilization trends of cardiometabolic drugs—particularly newer ones—and support the potential predictive capability of online search trends for near-real-time public health analyses.

## Data availability statement

The original contributions presented in the study are included in the article/Supplementary Material. Further inquiries can be directed to the corresponding author.

## Author contributions

PB and OD participated in the conception and drafted the manuscript. AR, RA, KJ, KN, JA, and MB revised subsequent drafts critically for important intellectual content. All authors contributed to the article and approved the submitted version.

## Funding

OD received support from National Institutes of Health grant T32 HL007227.

## Conflict of interest

MB reports grants from the National Institutes of Health, US Food and Drug Administration, American Heart Association, Bayer, and *Novo* Nordisk; Advisory Boards with Amgen, Sanofi, Regeneron, Novartis, Bayer, *Novo* Nordisk, Roche, Inozye, 89Bio, and Kaleido outside the submitted work.

The remaining authors declare that the research was conducted in the absence of any commercial or financial relationships that could be construed as a potential conflict of interest.

## Publisher's note

All claims expressed in this article are solely those of the authors and do not necessarily represent those of their affiliated organizations, or those of the publisher, the editors and the reviewers. Any product that may be evaluated in this article, or claim that may be made by its manufacturer, is not guaranteed or endorsed by the publisher.

## Abbreviations

CVD: cardiovascular disease
GLP-1 RA: Glucagon-like peptide-1 receptor agonist
SGLT2i: Sodium/glucose cotransporter-2 inhibitor
T2D: Type 2 diabetes.
