# Supplementary material for: Online searches for SGLT-2 inhibitors and GLP-1 receptor agonists correlate with prescription rates in the United States: An infodemiological study
Source: Front Cardiovasc Med. 2022 Jul 29;9:936651. doi: 10.3389/fcvm.2022.936651 (PMC9372305; doi:10.3389/fcvm.2022.936651)
Supplement: Supplementary file 1 [file Data_Sheet_1.docx]

**Supplementary Materials**

**Online Searches for SGLT-2 inhibitors and GLP-1 receptor agonists correlate with prescription rates in the United States: An Infodemiological Study**

Omar Dzaye MD MPH PhD^1†^, Philipp Berning MD^1,2†^, Alexander C. Razavi MD MPH PhD^1,3^, Rishav Adhikari^1^, Kunal Jha MD^1^, Khurram Nasir MD MPH MSc^4^, John W. Ayers MA PhD^5^, Martin Bødtker Mortensen MD PhD^1,6^, Michael J. Blaha MD MPH^1^

^1^ Johns Hopkins Ciccarone Center for the Prevention of Cardiovascular Disease, Johns Hopkins University School of Medicine, Baltimore, MD, United States

^2^ Department of Medicine, University Hospital Muenster, Muenster, Germany

^3^ Emory Center for Heart Disease Prevention, Emory University School of Medicine, Atlanta, GA, United States

^4^ Division of Cardiovascular Prevention and Wellness, Department of Cardiology, Houston Methodist DeBakey Heart & Vascular Center, Houston, TX, United States

^5^ Division of Infectious Diseases and Global Public Health, University of California, San Diego, CA, United States

^6^ Department of Cardiology, Aarhus University Hospital, Aarhus, Denmark

^†^ These authors share first authorship

**Supplementary Figure Legends**

**Supplemental Figure 1. ARIMA analysis for prescriptions of selected SGLT2i and GLP-1 RA.**

Trends in actual and expected prescription rates (per 10 million prescriptions) from January 2016 to December 2021 for dapagliflozin, empagliflozin, ertugliflozin, dulaglutide, semaglutide. Cut-off date for ARIMA analysis was January 01, 2021. Actual (dark blue) and expected (light blue) trends are shown.

**Supplemental Figure 2. Trends in prescriptions and online searches for Biguanide (metformin) and Sulfonylureas between 2016 and 2021 for the United States.**

(A) Online searches and (B) prescriptions from 2016 to 2021 for Biguanide (metformin) (red line) and Sulfonylureas (blue line) as monthly query fraction/prescriptions per 10 million searches/prescriptions for summarized brands names are shown. All data are representative for the United Sates.

**Supplemental Figure 1. ARIMA analysis for prescriptions of selected SGLT2i and GLP-1 RA.**

**
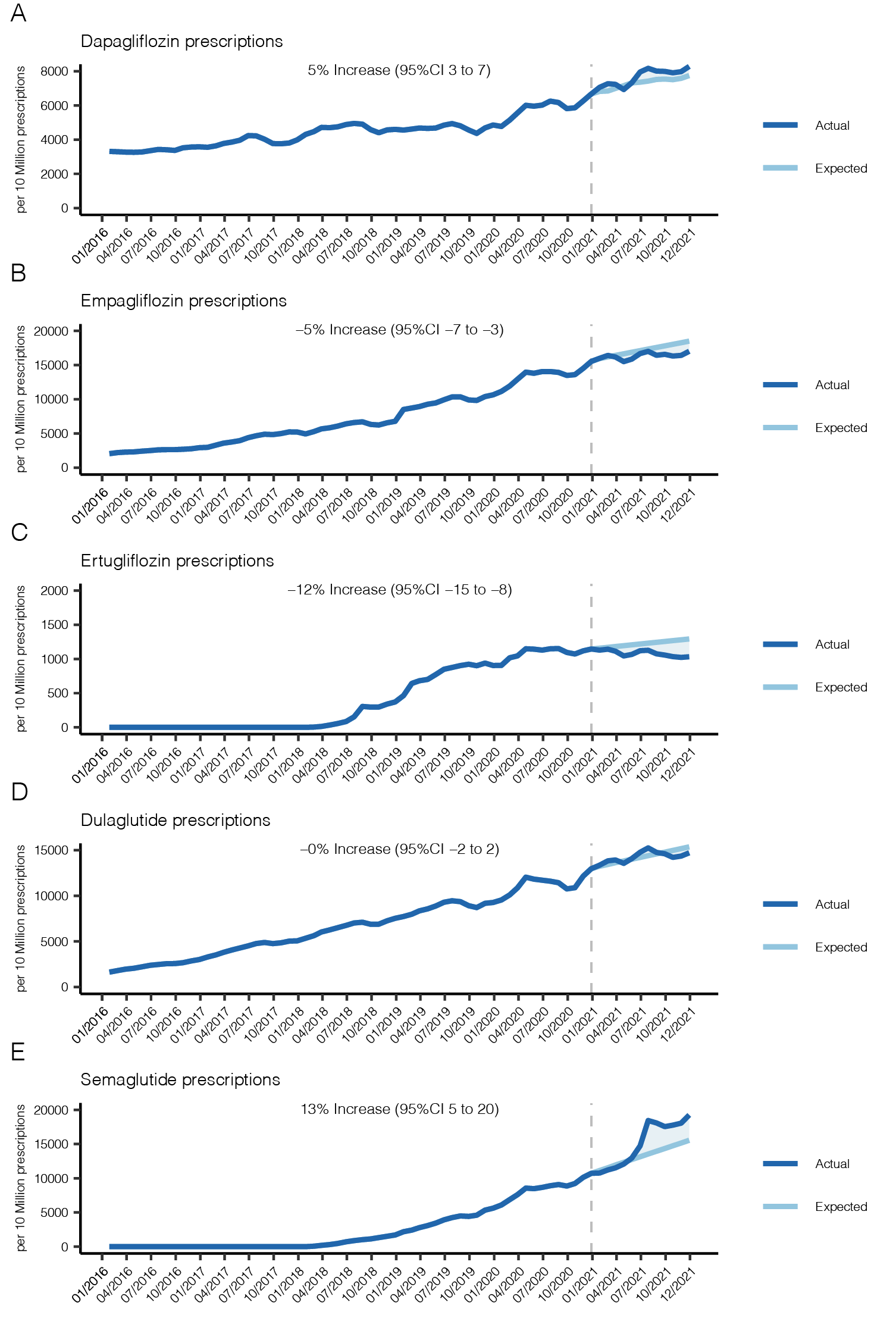
**

**Supplemental Figure 2. Trends in prescriptions and online searches for Biguanide (metformin) and Sulfonylureas between 2016 and 2021 for the United States.**

**Supplemental Table 1. Queried brand names and online search terms.**

| **Drug name** | **Prescription Data** | **Online Search Terms** |
| --- | --- | --- |
| *SGLT-2 inhibitors* |  |  |
| Dapagliflozin |  | Dapagliflozin |
|  | FARXIGA | FARXIGA |
|  | QTERN | QTERN |
|  | XIGDUO XR | XIGDUO XR |
| Canagliflozin |  | Canagliflozin |
|  | INVOKAMET | INVOKAMET |
|  | INVOKANA | INVOKANA |
| Empagliflozin |  | Empagliflozin |
|  | JARDIANCE | JARDIANCE |
|  | SYNJARDY | SYNJARDY |
|  | GLYXAMBI | GLYXAMBI |
|  | TRIJARDY | TRIJARDY |
| Ertugliflozin |  | Ertugliflozin |
|  | SEGLUROMET | SEGLUROMET |
|  | STEGLATRO | STEGLATRO |
|  | STEGLUJAN | STEGLUJAN |
| *GLP-1 receptor agonists* |  |  |
| Albiglutide |  | Albiglutide |
|  | TANZEUM | TANZEUM |
| Dulaglutide |  | Dulaglutide |
|  | TRULICITY | TRULICITY |
| Liraglutide |  | Liraglutide |
|  | VICTOZA | VICTOZA |
|  | SAXENDA | SAXENDA |
| Exenatide |  | Exenatide |
|  | BYDUREON | BYDUREON |
|  | BYETTA | BYETTA |
| Semaglutide |  | Semaglutide |
|  | OZEMPIC | OZEMPIC |
|  | RYBELSUS | RYBELSUS |
|  | WEGOVY | WEGOVY |
| *Biguanide* |  |  |
| Metformin |  |  |
|  | METFORMIN HCL | METFORMIN |
|  | METFORMIN ER (G) |  |
|  | METFORMIN ER (F) |  |
|  | GLUMETZA | GLUMETZA |
|  | RIOMET | RIOMET |
|  | GLUCOPHAGE | GLUCOPHAGE |
|  | FORTAMET ER | FORTAMET ER |
| *Sulfonylureas* |  |  |
| Glimepiride |  |  |
|  | GLIMEPIRIDE | GLIMEPIRIDE |
|  | AMARYL | AMARYL |
|  | GLIPIZIDE | GLIPIZIDE |
| Glipizide |  |  |
|  | GLIPIZIDE ER | GLIPIZIDE ER |
|  | GLIPIZIDE XL | GLIPIZIDE XL |
|  | GLUCOTROL XL | GLUCOTROL XL |
| Glyburide |  |  |
|  | GLUCOTROL | GLUCOTROL |
|  | GLYBURIDE | GLYBURIDE |
|  | GLYBURIDE MICRO | GLYBURIDE MICRO |
|  | GLYNASE PRESTAB | GLYNASE PRESTAB |
